# Association between the social support for mothers of patients with eating disorders, maternal mental health, and patient symptomatic severity: A cross-sectional study

**DOI:** 10.1186/s40337-020-00361-w

**Published:** 2021-01-06

**Authors:** Atsurou Yamada, Fujika Katsuki, Masaki Kondo, Hanayo Sawada, Norio Watanabe, Tatsuo Akechi

**Affiliations:** 1 Department of Psychiatry and Cognitive-Behavioral Medicine, Nagoya City University Graduate School of Medical Sciences, Nagoya, Japan; 2 Department of Psychiatric and Mental Health Nursing, Nagoya City University Graduate School of Nursing, 467-8601, 1 Kawasumi, Mizuho-cho, Mizuho-ku, Nagoya, Japan; 3 Department of Health Promotion and Human Behavior, Kyoto University Graduate School of Medicine/School of Public Health, Kyoto, Japan

**Keywords:** Caregiver, Depression, Eating disorders, Listening attitude, Loneliness, Psychological distress, Self-efficacy, Social support

## Abstract

**Background:**

Although caregivers of patients with eating disorders usually experience a heavy caregiving burden, the effects of social support on caregivers of patients with eating disorders are unknown. This study aimed to investigate how social support for mothers who are caregivers of patients with an eating disorder improves the mothers’ mental status and, consequently, the symptoms and status of the patients.

**Methods:**

Fifty-seven pairs of participants were recruited from four family self-help groups and one university hospital in Japan. Recruitment was conducted from July 2017 to August 2018. Mothers were evaluated for social support using the Japanese version of the Social Provisions Scale-10 item (SPS-10), self-efficacy using the General Self-Efficacy Scale, loneliness using the University of California, Los Angeles Loneliness Scale, listening attitude using the Active Listening Attitude Scale, family functioning using the Family Assessment Device, depression symptoms using the Beck Depression Inventory (Second Edition), and psychological distress using the Kessler Psychological Distress Scale. Patients were evaluated for self-esteem using the Rosenberg Self-Esteem Scale, assertion using the Youth Assertion Scale, and their symptoms using the Eating Disorder Inventory. We divided the mothers and patients into two groups based on the mean score of the SPS-10 of mothers and compared the status of mothers and patients between the high- and low-scoring groups.

**Results:**

High social support for mothers of patients with eating disorders was significantly associated with lower scores for loneliness and depression of these mothers. We found no significant differences in any patient scores based on mothers’ level of social support.

**Conclusions:**

For patients with eating disorders, social support for a caregiver cannot be expected to improve their symptoms, but it may help prevent caregiver depression and loneliness.

## Plain English summary

The effects of social support on caregivers of patients with eating disorders are unknown. This study aimed to investigate how social support for mothers who are caregivers of patients with an eating disorder improves the mothers’ mental status and, consequently, the symptoms and status of the patients. Fifty-seven pairs of participants were recruited from four family self-help groups and one university hospital in Japan. Mothers were evaluated for social support, self-efficacy, loneliness, listening attitude, family functioning, depression symptoms, and psychological distress. Patients were evaluated for self-esteem, assertion, and symptoms of eating disorder. We compared the status of mothers and patients between the high and low social support groups based on the mothers’ mean score on the Japanese version of the Social Provisions Scale-10 item. High social support for mothers was significantly associated with lower scores for loneliness and depression. There were no significant differences in any patient scores based on mothers’ level of social support. Social support for a caregiver of patients with eating disorders may help prevent caregiver depression and loneliness, but cannot be expected to improve patients’ symptoms.

## Background

Caregivers of patients with eating disorders usually experience a heavy caregiving burden, and are at risk of having poor mental health status [1] and a low quality of life [2]. The mean Hamilton Anxiety and Depression Scale score for 246 caregivers of patients with eating disorders was above the threshold for both anxiety and depression [3]. The level of depression and anxiety in caregivers, especially in mothers, was much higher than in a non-caregiver group [4]. Conversely, stress, anxiety and depression of caregivers of patients with eating disorders were all under the threshold on the Depression Anxiety Stress Scale [5]. According to a systematic review on eating disorders, eight studies that used the Hamilton Anxiety and Depression Scale reported that the levels of anxiety were high but the levels of depression were under the threshold [6]. Moreover, three studies using the Depression Anxiety Stress Scale reported that levels of anxiety and depression were normal and levels of stress were mild to severe [6]. Therefore, although there were variations in reports, caregivers’ anxiety and depression were generally high.

Previous studies have shown that providing social support for caregivers reduces their distress levels and enhances their mental health. Social support includes affective support and instrumental support. For example, a lack of affective support was significantly associated with worsened mental health status, and affective support was significantly associated with decreased severity of the patients’ symptoms from the family’s perspective [1]. Social support also includes spousal support. Among mothers of children with eating disorders, being married was shown to be a protective factor of caregiver burden, and the authors remarked that this protective function may due to spousal support [7]. However, there are also reports claiming that social support is not related to mental health or decreased psychological distress. For example, Dimitropoulos et al. reported that social support was related to family functioning but not to scores on the General Health Questionnaire [8]. Family functioning is the interaction of family members in a family environment, including attachment, parenting style, communication, and conflict [9]. Coomer et al. reported social support was not a predictor of burden or psychological distress among caregivers of people with eating disorders; rather, expressed emotion and caregivers’ needs were predictors of burden, and maladaptive coping was a predictor of psychological distress. However, they measured social support with Social Support Questionnaire 6, which is very general and affective in nature, and they stated that instrumental social support may be a predictor of burden and stress [10].

In summary, although previous reports have not agreed, social support may reduce psychological distress or improve mental health in caregivers of patients with eating disorders and it appears to be associated with lower accommodating behaviors and improved family functioning. Moreover, previous reports have also not agreed on whether social support reduces the severity of patients’ symptoms. We hypothesized that caregivers with more social support have less psychological distress and better family functioning and behaviors, and that patients have fewer symptoms than those with caregivers who have less social support.

Regarding caregivers, this study targets only mothers in consideration of the homogeneity of the subjects because it has been reported that mothers have a higher burden than fathers [7, 8, 11].

This study specifically aimed to investigate how social support for mothers who are caregivers of patients with eating disorders improves their mental status and, consequently, the symptoms and status of the patients. First, we aimed to investigate the association between mothers’ support status and distress and associated factors (e.g., self-efficacy, loneliness, listening attitude, depression, and psychological distress). The reason for assessing mother’s listening attitude in this study is that mothers with social support can afford to listen to the patient well, which may affect the patient’s symptoms. Second, we aimed to investigate the association between mothers’ support status and patients’ symptoms and associated factors (e.g., self-esteem, loneliness, self-assessed assertion, family functioning, and eating disorder symptoms) from the patients’ point of view.

## Methods

### Participants

Participants were patients with an eating disorder and their mothers. Our recruitment took place in four family self-help groups for patients with eating disorders and in one university hospital in Japan. Family self-help group is an organization in which families and patients with eating disorders participate and regularly hold meetings and lectures to support each other. Recruitment was conducted from July 2017 to August 2018. At the self-help group lectures, we invited participants from four locations: Hokkaido, Chiba, Fukui, and Nagoya, all in Japan. Each lecture was attended by approximately 50 family members of patients. The purpose of the study was explained via instructions attached to a questionnaire to patients and their mothers who were interested in the study. Subsequently, the questionnaires for mothers and patients were distributed at home via postal mail to those who were willing to take part in the survey. For outpatients and inpatients in the hospital, the researcher provided instructions to the patient and mother who were judged by the patient’s doctor to meet the inclusion criteria and were interested in the study. Next, the researcher handed them the self-report rating scale questionnaire. For mothers, the inclusion criteria were: aged between 30 and 85 years; mothers of patients who met the patient inclusion criteria. For patients, the inclusion criteria were: being a patient who had been confirmed by researchers to be clinically diagnosed with an eating disorder by a physician or psychiatrist in a hospital; aged between 16 and 50 years. We emphasize that we did not distinguish among specific eating disorders (e.g., anorexia nervosa and bulimia nervosa), but rather included patients with any type of eating disorder.

This study was approved by the Ethics Review Committee of Nagoya City University Graduate School of Medical Sciences, Japan (Ref: No 60-17-0001), and was conducted in accordance with the principles of the Declaration of Helsinki. Written informed consent was obtained from participants, and we have taken the necessary steps to ensure that all participants remained anonymous.

### Procedure

We gave participants a leaflet with an explanation of the purpose and procedure of the study. After receiving permission from patients’ mothers and their home addresses, we mailed the questionnaires to their homes. They were effectively enrolled in the study only when their completed questionnaires were returned. As described below, the questionnaire comprised questions on participants’ basic sociodemographic characteristics as well as questions from the Japanese version of the Social Provisions Scale-10 item (SPS-10), the University of California, Los Angeles Loneliness Scale (ULS), the Beck Depression Inventory (BDI-II), and the Kessler Psychological Distress Scale (K6), which were used to evaluate participants’ loneliness, depression, and anxiety levels.

### Outcome measures for mothers of patients with an eating disorder

#### Basic characteristics

In this study, we used the following to analyze mothers’ basic sociodemographic characteristics: (1) age, (2) patient’s eating disorder duration, (3) whether the patient (i.e., the daughter) receives medical care (if yes, for how many years), (4) whether the mother receives counseling (if yes, for how many years), (5) whether the mother has had an eating disorder, (6) whether the mother feels that she is cooperating with the father (if there is one) in handling the patient (yes, no, neither, not applicable), (7) whether the mother participates in a family self-help group (if yes, for how many years).

#### The social provisions Scale-10 item (SPS-10)

Mothers’ support status was evaluated using the Japanese version translated from the SPS-10, which is a shortened version of the Social Provisions Scale (SPS) created by Cutrona and Russell [12] and was developed by Iapichino et al. in Italian [13]. With permission from Iapichino et al., we created a Japanese version of the SPS-10 and described its development in another article [14]. The SPS-10 consists of 10 items and retains the following five of the six original SPS subscales: attachment (emotional support), social integration, reassurance of worth, reliable alliance (material support), and guidance. In the original SPS, each subscale has four items: two positively worded items describing the presence of a type of support, and two negatively worded items assessing the absence of a type of support. The SPS-10 has only negatively worded items, and the total score ranges from 10 to 40. A higher total score indicates stronger perceived provision of social support. The Cronbach’s α of the original Italian version was .809 [13], and in this study, it was α = .897.

#### General self-efficacy scale (GSES)

To assess mothers’ self-efficacy, we used the GSES developed by Sakano et al. [15, 16]. The GSES comprises three subscales and 16 items: aggressive behavior (7 items), anxiety about failure (5 items), and social positioning of ability (4 items). Each item was scored as 0 or 1. The higher the score, the higher the general self-efficacy. The Cronbach’s α of this study was α = .837.

#### University of California, los Angeles loneliness scale (ULS)

To assess mothers’ loneliness, we used the Japanese version (by Moroi) [17] of the ULS developed by Russell et al. [18]. The ULS is a 20-item scale, and each item was scored from 1 to 4; the higher the score, the stronger the loneliness. The Cronbach’s α of the Japanese version was .885 [19]. We obtained the author’s permission to use this scale. The Cronbach’s α of this study was α = .911.

#### Active listening attitude scale (ALAS)

To assess mothers’ listening attitude, we used the ALAS developed by Mishima et al. [20]. The ALAS comprises two subscales and 20 items: listening attitude (10 items) and listening skills (10 items). Each item was scored from 0 to 3. The higher the score, the better the listening attitude or skill. We obtained the authors’ permission to use this scale. The Cronbach’s α of this study was α = .855.

#### Beck depression inventory - second edition (BDI-II)

To assess mothers’ depression within the last 2 weeks, we used the Japanese version (by Kojima et al.) [21] of the BDI-II developed by Beck et al. [22]. The BDI-II is a 21-item self-report scale that assesses the presence and severity of depressive symptoms. Each item is rated on a 4-point scale ranging from 0 to 3; the higher the score, the more severe the depressive symptoms. As shown by two previous studies, the BDI-II is a reliable, internally consistent, and valid scale for assessing depression [22, 23]. The reliability and validity values of the Japanese version are excellent [24]. The Cronbach’s α of the Japanese version was 0.87 [21], and in this study, it was α = .931.

#### Kessler psychological distress scale (K6)

To assess mothers’ psychological distress, we used the Japanese version (by Furukawa et al.) [25] of the K6 developed by Kessler et al. [26]. The K6 is a 6-item self-reported scale that was developed to screen depression and anxiety disorders based on the Diagnostic and Statistical Manual of Mental Disorders Fourth Edition definitions, and it analyzes patients’ symptoms over the last 30 days. Moreover, it can also be used to quantify nonspecific psychological distress [26]. Items are rated from 0 to 4, and the total score ranges from 0 to 24; the higher the score, the more severe the psychological distress. Two independent studies have analyzed and confirmed the excellent validity of the K6 [26, 27], and a study on its Japanese version confirmed the same validity [25]. The Cronbach’s α of the original tool was 0.89 [26], and in this study, it was α = .869.

### Outcome measures of patients

#### Basic characteristics

In this study, we used the following to analyze patients’ basic sociodemographic characteristics: (1) age, (2) gender, (3) whether the patient receives counseling (if yes, for how many years), (4) whether the patient self-harms (if yes, how often), and (5) whether the patient feels that family members make many comments about the patient’s eating behaviors.

#### Rosenberg self-esteem scale (RSS)

To assess patients’ self-esteem, we used the Japanese version (by Abe et al.) [28] of the RSS [29]. The RSS is a 9-item scale, and each item was scored on a scale from 1 to 5; the higher the score, the higher the self-esteem. The reliability of the scale was α = 0.83 and the validity was confirmed [28]. The Cronbach’s α of this study was α = .920.

#### ULS

Patients’ loneliness was evaluated by the Japanese version of the ULS [17], the same scale used for mothers’ loneliness. The Cronbach’s α of this study was α = .935.

#### Youth assertion scale (YAS)

To assess the patients’ degree of assertion, we used the YAS developed by Tamase et al. [30]. The YAS comprises two subscales and 16 items: relationship formation (8 items) and persuasion and negotiation (8 items). Each item was scored from 1 to 5; the higher the score, the more assertive the person’s behaviors. The Cronbach’s α of the original study was α = .80 for relationship formation and .71 for persuasion negotiation; for this study, it was α = .832. The validity of the scale has also been confirmed [30].

#### Family assessment device (FAD)

To assess patients’ family functioning, we used the Japanese version (by Saeki et al.) [31] of the FAD developed by Epstein et al. [32]. The FAD is used to evaluate a family system based on the McMaster Model of Family Functioning, and it comprises seven subscales: problem solving (5 items), communication (6 items), roles (8 items), affective responsiveness (6 items), affective involvement (7 items), behavior control (9 items), and general function (12 items). In this regard, Ridenour et al. reported that best use of the FAD is using the General Functioning Subscale (12 items) as a summary score [33]. As the total number of question items was too large, we used only the General Functioning subscale: the General Functioning subscale had high internal consistency [33], and was used in another study [34]. Each item was scored from 1 to 4; the higher the score, the poorer the family functioning. The Cronbach’s α of the General Functioning subscales in the original study was α = .92 [32], in another study, α = .85 [34], and in this study, α = .925.

#### Eating disorder inventory (EDI)

To assess patients’ eating disorder symptom severity, we used the Japanese version (by Kiriike et al.) [35] of the EDI developed by Garner et al. [36]. The EDI is used to comprehensively evaluate the eating behaviors and psychological characteristics of patients with anorexia nervosa and bulimia nervosa, and comprises eight subscales with 64 items: drive for thinness (7 items), bulimia (7 items), body dissatisfaction (9 items), ineffectiveness (10 items), perfectionism (6 items), interpersonal distrust (7 items), interoceptive awareness (10 items), and maturity fears (8 items). Each item was scored from 0 to 3; the higher the score, the more severe the patient feels the symptoms of their eating disorder are. The Cronbach’s α for each subscale was α = 0.60–0.91, indicating high retest reliability [36].

### Statistical analysis

Descriptive data analysis was conducted by calculating median and mean scores and standard deviation. To find out if the status of mothers and patients differs between high and low social support groups, we divided the mothers into two groups based on the cutoff score for the SPS-10, which was 30 (i.e., if greater than or equal to 30, high-score group; if lower than 30, low-score group). As previous studies had used a cutoff SPS-10 score of 30 with valid results, the same point was used in this study [37]. Then, we compared mothers’ GSES, ULS, ALAS, BDI-II, and K6 scores as well as patients’ RSS, ULS, YAS, FAD, and EDI scores between the high- and low-scoring groups using unpaired t-tests, with *p* < 0.05 as significant. As the mother and patient were paired, the patient status was also compared between those groups to assess the impact of social support for the mother on the patient. Statistical analysis was performed using SPSS Statistics version 22.

## Results

### Participants’ characteristics

The study sample comprised 57 pairs: patients with an eating disorder and their mothers. The mean ages were 54.7 ± 6.6 years for mothers and 24.5 ± 6.9 years for patients. All patients were female. The patient’s eating disorder duration reported by her mother was 7.6 ± 5.3 years.

Table 1 summarizes the mothers’ baseline characteristics and scores. Baseline characteristics were obtained from 56 mothers. Most mothers received some support such as family self-help group, counseling, and/or cooperation with fathers. The mothers’ BDI-II was slightly higher than the normal Japanese sample [21], but their K6 was under the optimum cutoff of 12/13 for detecting serious mental illness [26]. The average scores for loneliness and the GSES were almost the same as those of the general population [16, 17].

**Table 1:** Baseline characteristics and scores for the instruments for mothers of patients with eating disorders

Questionnaire or Scale		n	%	mean	SD
Mother’s confirmation of the patient being under medical care (if yes, years)	Yes	43	75.4	6.5	6.0
	No	13	22.8		
Mother’s experience with counseling	Currently receiving	9	15.8		
	Received in the past	24	42.1		
	Never	23	40.4		
Mother’s past history of eating disorder	Yes	2	3.5		
	No	52	91.2		
	Unknown	2	3.5		
Mother’s feeling that the mother is cooperating with the father to handle the patient	Yes	31	54.4		
	No	6	10.5		
	Neither consent nor denial	15	26.3		
	Not applicable	4	7.0		
Mother’s experience with joining a family self-help group	Yes	38	66.7		
	Joined in the past	13	22.8		
	Never	4	7.0		
SPS-10		57		30.8	4.9
GSES		57		7.1	4.1
ULS		55		39.2	9.7
ALAS		56		36.1	7.0
BDI-II		57		13.9	10.4
K6		56		6.9	4.5

*SPS-10* Social Provisions Scale Japanese version, *GSES* General Self-Efficacy Scale, *ULS* University of California, Los Angeles Loneliness Scale, *ALAS* Active Listening Attitude Scale; Los Angeles Loneliness Scale, *BDI-II* Beck Depression Inventory-Second Edition, *K6* Kessler Psychological Distress Scale

Table 2 summarizes patient scores for each of the instruments used. The average RSS score was considerably lower than the average score of Japanese female university students [28]. The average YAS score was considerably lower than the average score of Japanese university students [30]. Regarding FAD, the total score for the 12 items of the General Functioning subscale was much higher than the average of Japanese university students and their families [31]. Unsurprisingly, the patient’s family functioning was considered to be impaired.

**Table 2:** Baseline characteristics and scores of the instruments applied to patients with eating disorders

Questionnaire or Scale			n	%	Mean	SD
Receiving counseling (if yes, years)	Yes		22	38.6		
	No		35	61.4		
Self-harms	Yes	Almost every day	0	0		
		About 2–3 times a week	2	3.5		
		About 2–3 times a month	1	1.8		
		Other	3	5.3		
	No		51	89.5		
Feeling that family members make many comments about the patient’s eating behaviors	Many comments		10	17.5		
	If anything, some		19	33.3		
	If anything, few		17	29.8		
	Few		11	19.3		
RSS			56		19.5	8.5
ULS			56		50.1	12.4
YAS			56		44.9	8.5
FAD			56		29.3	7.6
EDI-Total			57		92.1	37.5
EDI-drive for thinness			57		11.8	6.8
EDI-bulimia			57		8.5	6.9
EDI-body dissatisfaction			57		14.8	7.7
EDI-ineffectiveness			57		15.8	7.8
EDI-perfectionism			57		7.8	4.3
EDI-interpersonal distrust			57		8.3	4.1
EDI-interoceptive awareness			57		14.7	7.3
EDI-maturity fears			57		10.3	5.6

*ULS* University of California, Los Angeles Loneliness Scale, *RSS* Rosenberg Self-Esteem Scale, *YAS* Youth Assertion Scale, *EDI* Eating Disorder Inventory

### Comparison between the mothers’ high- and low-score groups for SPS-10

The mothers’ average score on the SPS-10 was 30.8 (SD = 4.9). High-score groups consisted of 34 mothers who scored greater than or equal to 30, and the low-score group consisted of 23 mothers who scored lower than 30. Table 3 shows a comparison of the mothers’ scores for each instrument. Mothers in the low-score group had significantly higher scores for the ULS (*p* < 0.01) and the BDI-II (*p* < 0.05) compared to those of the high-score group; namely, mothers who experienced less social support were shown to have greater loneliness and depressive symptoms. Mothers in the low-score group had higher scores for the K6 and lower scores for the ALAS and GSES than the high-score group, but there were no significant differences in the scores on the ALAS, K6, and GSES between the groups.

**Table 3:** Comparison of the scores on each of the instruments by two different groups of mothers

Scale	High-score mothers’ group^a^			Low-score mothers’ group^a^			*p*
	n	Mean	SD	n	Mean	SD	
GSES	34	7.4	4.1	23	6.7	4.1	.547
ULS	33	33.8	6.4	22	47.1	8.1	.000**
ALAS	34	36.9	8.2	22	34.8	4.5	.276
BDI-II	34	11.0	8.4	23	18.0	11.7	.011*
K6	34	6.4	4.1	22	7.7	5.1	.295

*ULS* University of California, Los Angeles Loneliness Scale, *ALAS* Active Listening Attitude Scale, *BDI-II* Beck Depression Inventory-Second Edition, *K6* Kessler Psychological Distress Scale, *GSES* General Self-Efficacy Scale

^a^The groups were divided based on the mother’s score being greater than/equal to or lower than 31 points on the Social Provisions Scale Japanese version (SPS-10)

Table 4 shows a comparison of patients’ scores by mothers’ group. Twenty-eight patients were in the high-scoring group and 27 were in the low-scoring group based on their mothers’ SPS-10 score. All patients answered the EDI, but for ULS, RSS, and YAS, one from the low-scoring group did not respond, and for the FAD, one from the high-scoring group did not respond. The patients’ average ULS and FAD scores were higher in the high-score mothers’ group, and the patients’ average RSS and YAS total scores were higher in the mothers’ low-score group: however, both pairs of scores were not significantly different. On the EDI subscale, there were no significant differences between the high-scoring mothers’ group and low-scoring mothers’ group. In general, the amount of support the mother felt did not seem to be significantly associated with patients’ symptoms.

**Table 4:** Comparison of patient scores for mothers in the high-score and low-score groups

Scale	High-score mothers’ group^a^			Low-score mothers’ group^a^			p
	n	Mean	SD	n	Mean	SD	
ULS	33	50.8	11.7	23	49.1	13.4	.604
RSS	33	18.2	7.8	23	21.5	9.2	.144
YAS	33	44.4	7.7	23	45.7	9.6	.555
FAD	33	29.7	6.8	23	28.8	8.8	.663
EDI-Total	34	92.0	34.8	23	92.2	41.9	.984
EDI-drive for thinness	34	11.8	6.5	23	11.7	7.2	.945
EDI-bulimia	34	8.4	6.9	23	8.7	7.0	.861
EDI-body dissatisfaction	34	14.8	7.3	23	14.8	8.3	.999
EDI-ineffectiveness	34	16.4	7.6	23	14.9	8.2	.475
EDI-perfectionism	34	7.4	4.2	23	8.4	4.5	.386
EDI-interpersonal distrust	34	8.2	3.5	23	8.5	4.9	.757
EDI-interoceptive awareness	34	14.6	7.0	23	14.8	7.8	.893
EDI-maturity fears	34	10.3	5.5	23	10.2	6.0	.945

*ULS* University of California, Los Angeles Loneliness Scale, *RSS* Rosenberg Self-Esteem Scale, *YAS* youth assertion scale, *EDI* Eating Disorder Inventory, *FAD* Family Assessment Device

^a^The groups were divided based on the mother’s score being greater than/equal to or lower than 31 points on the Social Provisions Scale Japanese version (SPS-10)

## Discussion

In this study, we investigated how social support for mothers who are caregivers of patients with eating disorders is associated with maternal mental status and patients’ symptomatic severity. Our results showed that mothers who felt that they received higher levels of social support felt less isolated and less depressed than those that felt that they received lower levels of social support. However, there were no significant differences in listening attitude, self-efficacy, or psychological distress between mothers who felt that they received high levels of social support and those who felt that they received low levels of social support.

Coomber et al. reported that social support was not a significant predictor of burden or psychological distress among caregivers of people with eating disorders [10]. In our study, mothers in the low-score group for the SPS-10 had significantly higher scores for the BDI-II than did the high-score group for the SPS-10, but there were no significant differences in the scores for the K6 evaluating psychological distress. Although there were distinct differences in scales and measurement methods between our study and previous studies, our results were consistent with the finding that social support did not predict psychological stress. Dimitropoulos et al. reported that decreased social support predicted increased family dysfunction but not psychological distress, and that family dysfunction was correlated with high levels of burden in caregivers of patients with anorexia nervosa [8]. This is similar to the results of our study, in which there was no significant difference in the scores of K6 between mothers receiving high levels of social support and those receiving low levels of social support. However, as shown in Table 4, in our study there were no differences in patients’ family functioning between mothers receiving high levels of social support and those receiving low levels of social support. We believe the difference between our results and those of the cited study may be due to family functioning in our study being based on patients’ self-reports, not the mothers’. Martin et al. reported that being married was a protective factor with regard to caregiver burden for mothers, but their research merely investigated marital status, not whether mothers felt supported [7]. Our study investigated mothers’ feelings of support, but did not specify who provided it. In our study, the percentage of mothers (from both groups) who could cooperate with fathers was 54.4%, suggesting that they might be receiving support from fathers.

Therefore, there were no differences in the level of psychological stress between mothers who are caregivers of patients with eating disorders who feel that they receive substantial social support and those who do not. However, our study findings showed that their loneliness and depression might be alleviated by social support, as revealed by their scores.

Additionally, our results showed that there were no differences in patients’ loneliness, self-esteem, degree of assertion, family functioning, or eating disorder symptom severity levels when comparing patients who were cared for by mothers receiving high levels of social support and those who were cared for by mothers who received low levels of social support.

Contrary to our findings, Ohara et al. have shown that mothers receiving social support provide self-assessed reports stating that their care recipients have lower symptom severity [1]. We believe the difference between our results and those of the cited study may owe to symptomatic severity in our study being based on *patients’* self-reports, not their *mothers’*. In corroboration with our findings, Rhind et al.’s study found no association between caregivers’ social support and patients’ self-assessments of their eating disorder symptom severity [11].

We believe our results and the similar results of this cited study may be owing to mothers who receive more social support being more likely to underestimate their care recipients’ symptomatic severity, which may not concur with patients’ self-assessments on the same topic. Thus, regardless of whether mothers who are caregivers for patients with eating disorders feel that they receive social support, there are no differences in the patients’ view of loneliness, severity of symptoms, assertions, and family functioning do not appear to change significantly.

### Study strengths

Our study had several strengths: first, it targeted not only the mothers who are caregivers of patients with eating disorders, but also the patients themselves. While most previous studies have evaluated patients’ symptoms from the caregivers’ point of view [1, 7, 10, 38] with the exception of one study [11], ours provided patients’ self-assessments regarding their symptoms. This methodological approach made it possible for our analyses to demonstrate whether caregivers’ social support was associated with patients.

Second, participants comprised both caregivers of patients who are outpatients at a hospital and caregivers who participate in a local family association in the community (not the hospital). It represents a wider range of patients, including those who do not need medical care and those who refuse medical care, as well as those who receive medical care.

Third, this study measured factors other than caregivers’ social support (e.g., self-efficacy, loneliness, and depression) as well as patient factors (e.g., patients’ loneliness and degree of assertion). As a result, we were able to show that social support for caregivers was related to low isolation and low depression of caregivers, but not to psychological stress, self-efficacy, or listening attitudes of caregivers. Furthermore, we were able to show that social support for caregivers was not related to the severity of symptoms felt by the patient. Given that there is no established model for how social support affects the depression and care burden of mothers who are caregivers of patients with eating disorders, we hope that the results of this study will help clarify the issue.

### Limitations

First, this was a cross-sectional study. Therefore, our results alone cannot show causal relationships; that is, our results do not allow for the final conclusion that social support does not affect patients’ symptoms. It is merely demonstrated that maternal social support is not *associated with* a reduction in patient symptoms.

Second, we had a small sample size. This may have resulted in the small effects we observed, subsequently not allowing for the detection of any significant differences among the studied variables.

Third, we did not identify or differentiate any subgroups of eating disorders, and the participating patients’ age range was wide. Some previous studies targeted only anorexia patients [1, 8], and another restricted its sample to adolescents [39]. Moreover, it has been reported that compared to caregivers of bulimia patients, caregivers of anorexia patients experienced a higher amount of problems in three areas: “not enough information on rehabilitation,” “problems caused by relapses or crises,” and “depression, anxiety, burn-out, physical illness of the carer [40].” Thus, we believe that future studies should distinguish patients by type of eating disorder, as different types seem to evoke different outcomes for the caregivers.

Fourth, this study measured mothers’ social support through the SPS-10, but previous studies have used other scales; for example, social support was measured by the Social Provisions Scale [8], the Social Network Questionnaire [1], the Social Support Questionnaire [10] and the Oslo three-item social support [11]. Therefore, it is difficult to directly compare the content and amount of social support with other studies.

Fifth, we measured patients’ eating disorder symptom severity through the EDI, but we did not check the patient’s diagnosis of eating disorders or overall severity such as disorders in daily life or body weight. Therefore, this study may only evaluate one aspect of the patient’s symptoms.

### Recommendations

Based on these limitations, we believe that future studies are warranted to clarify the mechanism by which social support affects patients with eating disorders and their caregivers. Additionally, future studies on the topic should have larger samples, subdivide eating disorders by type (e.g., anorexia versus bulimia), divide patients by age, divide the social support construct into more subfactors, and include caregivers who do not receive appropriate amounts of support. Finally, further relevant factors should be posited, so as to allow for the development of a model that delineates the mechanisms behind these studied variables. This model may enable practitioners to improve patients’ symptoms and maternal/caregivers’ depression through the provision of interventions tailored to patients with eating disorders and to their caregivers.

## Conclusions

This study aimed to investigate how social support for mothers who are caregivers of patients with eating disorders improves their mental status and, consequently, the symptoms and status of the patients. High social support for mothers of patients with eating disorders was significantly associated with lower loneliness and depression of the mothers. However, there were no significant differences in the symptoms and status of the patients by comparing based on mothers’ level of social support.

## Data Availability

The datasets used and/or analyzed during the current study are available from the corresponding author on reasonable request.

## Abbreviations

ALAS: Active Listening Attitude Scale
BDI-II: Beck Depression Inventory
EDI: Eating Disorder Inventory
FAD: Family Assessment Device
GSES: General Self-Efficacy Scale
K6: Kessler Psychological Distress Scale
RSS: Rosenberg Self-Esteem Scale
SPS: The Social Provisions Scale
SPS-10: The Social Provisions Scale-10 item
ULS: University of California, Los Angeles Loneliness Scale
YAS: Youth Assertion Scale
